# The Effects of Fenugreek Extract on Growth Performance, Serum Biochemical Indexes, Immunity and NF-κB Signaling Pathway in Broiler

**DOI:** 10.3389/fvets.2022.882754

**Published:** 2022-06-23

**Authors:** Hui Huang, Xia Wang, Ling Yang, Wenxiang He, Tiantian Meng, Ke Zheng, Xin Xia, Yingjun Zhou, Jianhua He, Chunming Liu, Shengwen Zou, Dingfu Xiao

**Affiliations:** ^1^College of Animal Science and Technology, Hunan Agricultural University, Changsha, China; ^2^Yiyang Vocational and Technical College, Yiyang, China; ^3^Geneham Pharmaceutical Co., Ltd., Changsha, China; ^4^College of Xiangya Pharmaceutical Sciences, Central South University, Changsha, China; ^5^Hunan Wenfeng Agricultural and Animal Husbandry Technology Co., Loudi, China

**Keywords:** fenugreek seed extract, broiler, immunity, production performance, NF-κB signaling pathway

## Abstract

In order to study the regulation of Fenugreek seed extract (FSE) on the immunity of broilers, and explore the appropriate amount of FSE in broilers' production, 1-day-old yellow feather broilers with a total of 420 birds were randomly allocated into seven treatments. Each treatment had six replicates, with 10 birds per replicate. The two control groups were the basic fodder group fed with basal diet and the bacitracin zinc group added 30 mg/kg bacitracin zinc to the basal diet. Experimental groups included five levels of FSE (50, 100, 200, 400, and 800 mg/kg FSE to the basal diet, respectively). The pre-test period was 7 days and the formal test lasted for 56 days. The results showed that the average daily gain (ADG) of 50 and 800 mg/kg FSE groups was significantly increased (*P* < 0.01), and the feed to gain ratio (F/G) of FSE groups was significantly decreased (*P* < 0.01) compared with the basic fodder and the bacitracin zinc groups. Compared with the basic fodder group, the serum total cholesterol (TC) content in the FSE groups was significantly decreased (*P* < 0.05), the serum low density lipoprotein cholesterol (LDL-C) content of 50, 100, and 800 mg/kg FSE groups was significantly lower than that of the basic fodder group (*P* < 0.05). Compared with the basic fodder and bacitracin zinc groups, the serum immunoglobulins (IgG, IgM, IgA) content of 100 and 200 mg/kg FSE groups were significantly increased (*P* < 0.05). Compared with the bacitracin zinc group, the serum interleukins (IL-1, IL-10) content of 400 mg/kg FSE group were significantly increased (*P* ≤ 0.05), and the serum interferon-γ (IFN-γ) content of 100 and 200 mg/kg FSE groups was significantly increased (*P* < 0.05). Compared with the basic fodder group, the lower doses (0–400 mg/kg) of FSE had no significant effect on the mRNA expression of toll-like receptors 4/ myeloid differentiation factor 88/ nuclear factor-κB (TLR4/MyD88/NF-κB) signaling pathways (*P* > 0.05). The 800 mg/kg FSE treatment group significantly increased the expression levels of nuclear factor-κB (NF-κB) mRNA in the spleen of broilers (*P* < 0.05). The zinc bacitracin group significantly increased the expression levels of myeloid differentiation factor 88 (MyD88) and nuclear factor-κB (NF-κB) mRNA (*P* ≤ 0.05). The results showed that FSE could promote the secretion of immunoglobulins, regulate the body's cytokines, and have a positive effect on immunity in broilers. Furthermore, the recommended supplement of FSE is 100 mg/kg in the broiler diet.

## Introduction

Antibiotics are widely added in feed because they are beneficial to the development and disease resistance of livestock and poultry. However, the long-term use of antibiotics makes animals resistant to antibiotics, while leaving antibiotics in animal products, adversely affecting human health (1, 2). Since July 1, 2020, banning the use of antibiotics in livestock and poultry nutrition in China, plant secondary metabolites and their derivatives have attracted a lot of attention for their potential role as alternatives for antibiotics. The research of plant extracts replacing antibiotics has become a hot spot (3). Fenugreek is a famous medicinal plant. The plant, about 60 cm tall, has light yellow triangular-shaped flowers and trilobal-shaped leaves. It is widely used in medicine as one type of plant extracts that have been frequently studied in recent years. Fenugreek is a multifunctional herb belonging to the family of fabacecae which is known for its antifungal, antiviral, anticarcinogenic, antidiabetic and antimicrobial properties (4). Fenugreek extract mainly refers to plant extracts from fenugreek seeds that have a certain beneficial effect on the animal body. It is mainly rich in polyphenols, flavonoids, saponins, polysaccharides and alkaloids, and other nutrients and chemical components, which have positive effects on livestock and poultry such as promoting growth and improving immunity (5). Some researchers have reported that fenugreek can stimulate humoral and cellular immune mechanisms (6, 7) and regulate body immunity (8–11). However, others have noticed that the use of fenugreek in layer diets in amounts of 1.00 and 2.00% has a negative influence on the egg production. The objective of the present study was to confirm previous work on dietary supplementation of fenugreek extract on growth performance, immune organ indexes, blood parameters and NF-κB signaling pathway in broilers, and explore whether fenugreek extract can replace antibiotics as a new feed additive and be applied to broiler production. Moreover, the study is helpful for finding out the appropriate concentration of fenugreek extract that is beneficial to the growth of broiler and the health of broiler production. It provides new ideas and methods for improving the growth of broiler and the health of broilers by means of nutritional regulation in production and also provides a reference for the research of FSE in improving the quality and health of chickens.

## Materials and Methods

### Experimental Materials and Diet Composition

The fenugreek extract used in the experiment contained 50% polysaccharides, 10% flavonoids, 15% saponins, 2% alkaloids and other ingredients, and was provided by Hunan Jinhan Pharmaceutical Co., Ltd. The fenugreek was extracted by water, concentrating the filtrate after two times of reflux extraction and then spray-drying into powder.

### Animals and Experimental Design

The animal experiment was approved by the Institutional Animal Care and Use Committee of Hunan Agricultural University, Hunan, China.

In this study, a total of 420 1-day-old yellow feather broilers with similar body weight (BW)(55.30 ± 0.10 g) were randomly allocated into seven treatments. Each treatment had six replicates, with 10 birds per replicate. Five concentrations of FSE experimental groups were added 50, 100, 200, 400, and 800 mg/kg FSE to the basal diet, respectively. The trial period lasted 63 days. The first 7 days were a pre-test period lasted for the broiler to adapt to the diets, and the formal trial period lasted for 56 days. According to the standard nutritional requirement of broilers (Agricultural industry standard of the people's Republic of China—chicken breeding standard NY/T33-2004), the basic diet formula of the formal trial period was divided into two periods (1–28, 29–56 days). The composition and nutritional level of basic diet and premix were shown in Tables 1, 2, respectively. The experiment was conducted by a 3-layer cage with natural ventilation, each cage was a replicate of 10 chickens, and the 6 replicates of each treatment were divided into two 1–3 layers, which were evenly distributed in each position of the chicken house. Birds received 24 h of light until day 4; then, light duration was decreased to 18 h for the remainder of the trial. The temperature gradually decreased from 32°C on day 1 to 20°C on day 27, and the humidity was maintained at 50–65%. The experimental chickens had free diets and water, and the experiment routine immunization and disinfection.

**Table 1:** Composition and nutrient levels of basal diets (DM basis) %.

**Ingredients**	**1 to**	**28 to**	**Nutrient**	**1 to**	**28 to**
	**28 days**	**56 days**	**levels^**b)**^**	**28 days**	**56 days**
	**of age**	**of age**		**of age**	**of age**
Corn	56.55	58.65	ME/(MJ/kg)	12.20	12.46
Soybean meal	36.10	33.40	CP	20.13	19.17
Soybean oil	3.00	3.50	Lys	1.01	0.97
CaHPO_4_	1.80	1.90	Met	0.43	0.41
Limestone	1.00	1.00	Ca	1.09	1.11
NaCl	0.30	0.30	AP	0.52	0.53
Cholone chloride	0.15	0.15			
DL-Met	0.10	0.10			
Premix^a)^	1.00	1.00			
Total	100.00	100.00			

^a)^* The premix same as Table 2*.

^b)^* Nutrient levels were all calculated values*.

**Table 2:** Nutrient level of premix (provided per kg of diet).

**Nutrient levels^**2)**^**	**Content**	**Nutrient levels^**2)**^**	**Content**
Fe	90 mg	VB_6_	1.5 mg
Mn	90 mg	VB_12_	10 μg
Zn	50 mg	VA	5,000 IU
Cu	10 mg	VD	3,500 IU
Co	0.4 mg	VE	10 IU
I	0.4 mg	nicotinic acid	35 mg
Se	0.2 mg	folic acid	0.8 mg
VB_2_	6.0 mg	pantothenic acid	12 mg
VB_1_	1.5 mg	biotin	0.8 mg

^2)^* Nutrient levels were all calculated values*.

### Sample Collection and Analysis

#### Growth Performance

At day 56 of the experiment, broilers were weighed on an empty stomach (fasting for 12 h). The average daily gain (ADG), average daily feed intake (ADFI) and feed conversion ratio (Feed/Gain, F/G) of the birds were determined.


$$\begin{array}{l} \text{Average daily feed intake (ADFI)}\\ \;=\text{total feed intake}/\left(\text{test days}\times \text{total number of test broilers}\right) \end{array}$$

$$\begin{array}{l} \;\text{Average daily gain (ADG)}\\ \;=\left(\text{final body weight}-\text{initial body weight}\right)/\text{test days} \end{array}$$

$$\begin{array}{l} \;\text{Feed to gain ratio (F/G)}\\ \;=\text{average daily feed intake / average daily gain} \end{array}$$

#### Immune Organ Indexes

At day 56 of the experiment, healthy broilers weighing close to the average weight from each repetition were randomly selected and weighed after 12 h feed withdrawal and immediately slaughtered after exsanguination. The thymus, spleen and bursa of Fabricius were separated after dissection and weighed. Immune organ indexes were calculated according to the change of the immune organ weight. The spleen, thymus, and bursa of Fabricius indexes were calculated.


$$\begin{array}{lll} \text{Immune organ indexes (\%)} & = & 100\times \text{Immune organ weight (g)}\\ /\text{body weight (g)} \end{array}$$

#### Serum Biochemical Indexes

Blood samples (20 mL) were taken from the wing vein and centrifuged at 3,500 r/min for 10 min at 4°C after standing at 37°C for 1 h to prepare serum. The separated serum was frozen at −20°C pending analysis. The content of total protein (TP), albumin (ALB), triglycerides (TG), total cholesterol (TC), high density lipoprotein cholesterol (HDL-C), low density lipoprotein cholesterol (LDL-C), glucose (GLU), alanine transaminase (ALT), aspartate transaminase (AST), urea nitrogen (BUN) and creatinine (CREA) were measured with commercial kits (Jiancheng Bioengineering Institute) using an Mindray BS200 automatic biochemical analyzer (12).

#### Serum Immune Indexes

Individual serum samples were detected for the levels of serum immunoglobulin (IgA, IgG, IgM) and cytokines (IL-1, IL-2, IL-6, IL-10, INF-γ, and TNF-α) by ELISA using test kits purchased from Jiangsu Yutong Biological Technology Co., Ltd. (Yancheng, China), following the manufacturer's instructions (13).

#### Quantitative Real-Time PCR Analysis

The mRNA expression of nuclear factor-κB (NF-κB), toll-like receptor 4 (TLR4), myeloid differentiation factor 88 (MyD88), interferon-γ (IFN-γ), interleukin-1 (IL-1), interleukin-10 (IL-10), and TNF receptor associated factor 6 (TRAF6) in the broilers' spleen was quantified by quantitative real-time PCR. GAPDH was used as the reference gene to normalize the gene expression data. According to the gene sequence retrieved by Gene in NCBI, the primers were designed and synthesized by Shenggong Bioengineering (Shanghai) Co., Ltd. The sequence is shown in Table 3.

**Table 3:** Primer sequences for quantitative real-time PCR analysis.

**Gene**	**Primer Sequence (5^**′**^–3^**′**^)**	**Product/bp**
GAPDH-F	GTGCTGGTAGAGGGATGCTTATGC	116
GAPDH-R	CGGCAGGTCAGGTCAACAACAG	
TLR4-F	GCCATCCCAACCCAACCACAG	121
TLR4-R	CCACTGAGCAGCACCAATGAGTAG	
MyD88-F	CCACAACACCACGGACACAGC	118
MyD88-R	CAGCAGCCAGTCAAGCAGAAGG	
TRAF6-F	TAGCACGCAGCCTTGAGTTAGTTG	117
TRAF6-R	GCATCAGCAGTGGCAGAAGTAGAG	
NF-κB-F	CAGCCCATCTATGACAACCG	151
NF-κB –R	CAGCCCAGAAACGAACCTC	
IFN-γ-F	CAAGCTCGTGCCTGGTTCCTG	81
IFN-γ-R	CAAGGGTGGGAGGTGCGTTTG	
IL-1-F	ACCCGTTGACCCGTCCCTTC	124
IL-1-R	CTTGTAGCCCTTGATGCCCAGTG	
IL-10-F	GTCGCTCCTTCTCCTTCTGTTTCG	144
IL-10-R	TGTTGTCTCGCAGCAAGCAGTG	

### Statistical Analysis

**Figure 1:**
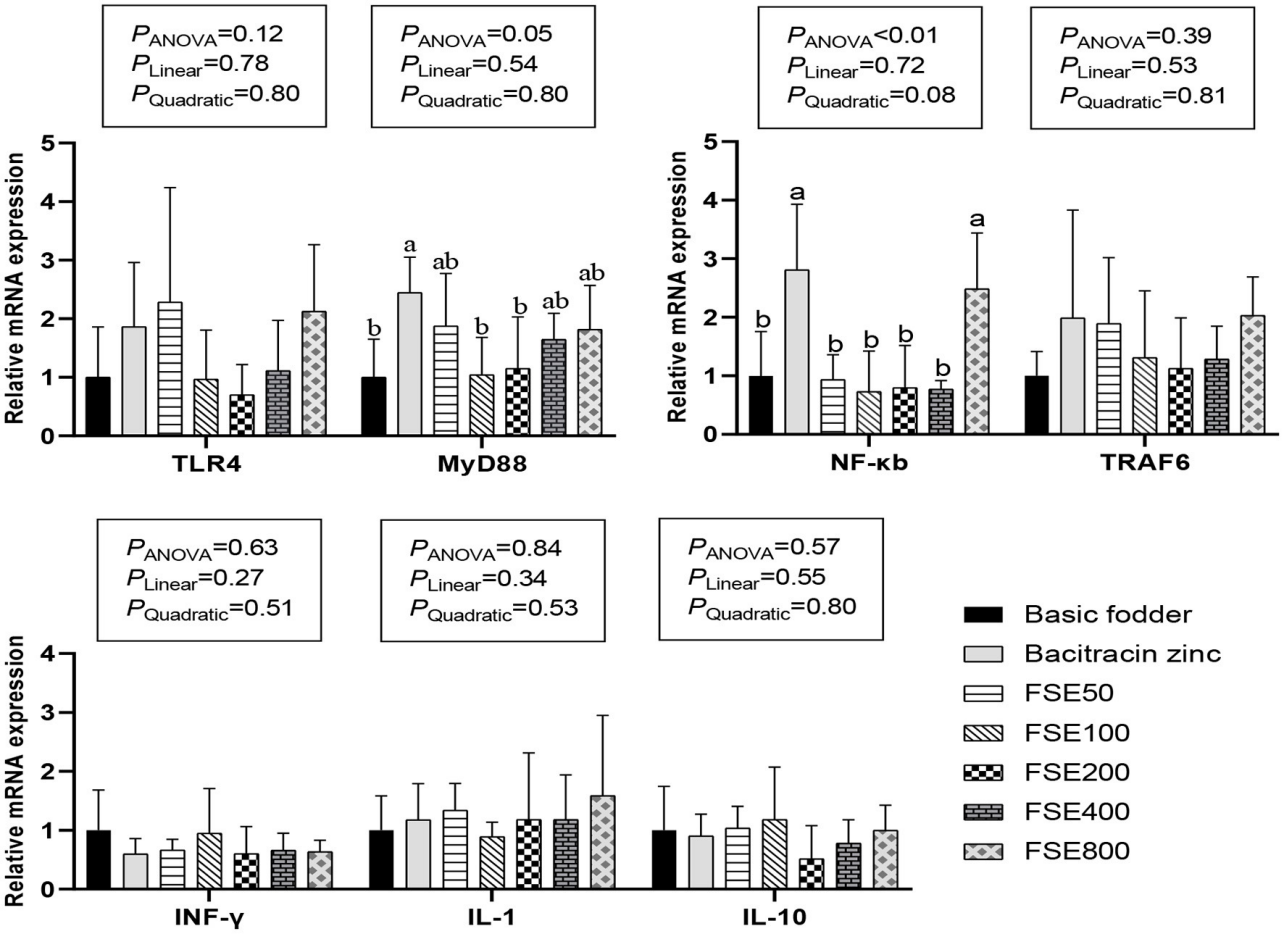
Effects of fenugreek seed extract on gene expression of TLR4/MyD88/NF-κB signaling pathway in spleen of broilers.

The statistical methods were used to analyze the data in Tables 4–8; Figure 1. All of the data were expressed as the mean ± standard deviation (SD) and were analyzed statistically by one-way ANOVA and Duncan multiple comparison of variance using SPSS 21.0.The linear and quadratic effects of dietary FSE supplementation levels were determined by polynomial contrasts. The differences were considered to be significant at *P* ≤ 0.05.

**Table 4:** Effect of fenugreek seed extract on growth performance of broilers^1^.

**Items**	**Basic**	**Bacitracin**	**FSE(mg/kg)**					***P*** **-value**		
	**fodder**	**zinc**	
			**50**	**100**	**200**	**400**	**800**	**ANOVA**	**Linear**	**Quadratic**
Initial BW (g)	55.25 ± 2.55	55.38 ± 2.59	55.30 ± 2.53	55.30 ± 2.59	55.32 ± 2.57	55.35 ± 2.60	55.25 ± 2.51	0.94	0.99	0.99
Final BW (g)	1083.09 ± 43.64^c^	1080.9 ± 28.60^c^	1199.71 ± 62.52^a^	1161.88 ± 50.66^a^	1143.76 ± 42.97^ab^	1116.29 ± 26.90^bc^	1202.44 ± 65.87^a^	<0.01	<0.01	0.01
ADFI (g/d)	48.27 ± 2.26	51.18 ± 2.19	50.29 ± 2.12	46.66 ± 4.79	48.85 ± 3.95	47.21 ± 1.73	50.24 ± 3.78	0.15	0.63	0.72
ADG (g)	20.58 ± 0.85^c^	20.58 ± 0.90^c^	22.84 ± 1.19^ab^	22.05 ± 1.44^abc^	21.84 ± 1.24^abc^	21.51 ± 0.69^bc^	23.13 ± 1.60^a^	<0.01	<0.01	0.01
F/G	2.35 ± 0.05^b^	2.49 ± 0.01^a^	2.20 ± 0.09^cd^	2.11 ± 0.11^d^	2.24 ± 0.10^c^	2.19 ± 0.04^cd^	2.17 ± 0.04^cd^	<0.01	<0.01	<0.01

*Different lowercase letters with the same column date meant significant difference (P ≤ 0.05)*.

^1^* Each value represents the mean of 6 replicate pens*.

**Table 5:** Effect of fenugreek seed extract on immune organ index of broilers (%)^1^.

**Items**	**Basic fodder**	**Bacitracin zinc**	**FSE(mg/kg)**					***P*** **-value**		
			**50**	**100**	**200**	**400**	**800**	**ANOVA**	**Linear**	**Quadratic**
Thymus index	1.31 ± 0.80	1.24 ± 0.40	0.94 ± 0.54	1.35 ± 0.66	1.09 ± 0.85	0.74 ± 0.43	0.65 ± 0.54	0.34	0.03	0.08
Spleen index	1.82 ± 0.19	1.70 ± 0.29	1.98 ± 0.27	1.82 ± 0.23	1.93 ± 0.55	1.78 ± 0.43	2.05 ± 0.51	0.73	0.32	0.61
Bursa of Fabricius index	1.26 ± 0.71	1.39 ± 0.83	1.04 ± 0.83	1.64 ± 0.60	1.37 ± 0.74	1.35 ± 0.42	0.88 ± 0.17	0.60	0.97	0.99

*Different lowercase letters with the same column date meant significant difference (P ≤ 0.05)*.

^1^* Each value represents the mean of 6 replicate pens*.

**Table 6:** Effects of fenugreek seed extract on serum biochemistry in broilers^1^.

**Items**	**Basic fodder**	**Bacitracin zinc**	**FSE(mg/kg)**					***P*** **-value**		
			**50**	**100**	**200**	**400**	**800**	**ANOVA**	**Linear**	**Quadratic**
TP (g/L)	35.89 ± 2.06	34.65 ± 4.46	35.71 ± 2.39	36.32 ± 1.97	35.37 ± 2.40	36.13 ± 3.71	32.09 ± 5.92	0.44	0.88	0.61
ALB (g/L)	11.51 ± 1.00	11.11 ± 1.07	10.51 ± 1.50	10.65 ± 0.86	10.97 ± 0.52	10.86 ± 1.12	10.87 ± 0.65	0.73	0.52	0.48
TG (mmol/L)	0.49 ± 0.16	0.33 ± 0.21	0.35 ± 0.14	0.31 ± 0.14	0.40 ± 0.21	0.32 ± 0.16	0.24 ± 0.13	0.28	0.05	0.14
TC (mmol/L)	3.95 ± 0.49^a^	3.60 ± 0.39^ab^	3.36 ± 0.29^b^	3.42 ± 0.23^b^	3.66 ± 0.24^ab^	3.33 ± 0.40^b^	3.46 ± 0.11^b^	0.04	<0.01	0.02
HDL-C (U/g)	2.35 ± 0.33	2.26 ± 0.35	2.40 ± 0.22	2.41 ± 0.13	2.30 ± 0.25	2.04 ± 0.33	2.12 ± 0.32	0.23	0.06	0.07
LDL-C (U/g)	1.15 ± 0.36^a^	0.82 ± 0.25^bc^	0.60 ± 0.05^c^	0.72 ± 0.17^bc^	1.01 ± 0.19^ab^	1.00 ± 0.27^ab^	0.80 ± 0.25^bc^	0.01	0.63	0.18
GLU (mmol/L)	11.53 ± 0.65^b^	12.63 ± 1.02^ab^	12.32 ± 0.73^ab^	12.57 ± 1.12 ^ab^	13.23 ± 1.31^a^	12.70 ± 0.99^ab^	13.61 ± 0.78^a^	0.04	0.11	0.13
ALT (U/L)	12.12 ± 2.31	14.41 ± 3.91	12.86 ± 3.65	13.05 ± 2.41	14.97 ± 9.92	11.88 ± 2.45	12.44 ± 3.37	0.58	0.77	0.52
AST (U/L)	203.25 ± 32.32	237.28 ± 32.96	231.07 ± 20.83	233.18 ± 24.41	238.00 ± 29.99	226.59 ± 10.11	231.41 ± 17.23	0.40	0.68	0.91
BUN (mmol/L)	0.28 ± 0.10	0.46 ± 1.40	0.39 ± 0.20	0.43 ± 0.18	0.47 ± 0.27	0.61 ± 0.11	0.38 ± 0.25	0.19	0.12	0.12
CREA (umol/L)	36.34 ± 25.38	18.73 ± 13.32	22.71 ± 8.88	26.69 ± 15.93	19.01 ± 5.98	15.56 ± 8.49	21.70 ± 12.37	0.27	0.22	0.47

*Different lowercase letters with the same column date meant significant difference (P ≤ 0.05)*.

^1^* Each value represents the mean of 6 replicate pens*.

**Table 7:** Effects of fenugreek seed extract on serum immunoglobulin in broilers^1^.

**Group**	**IgG**	**IgM**	**IgA**
Basic fodder	818.25 ± 283.79^bc^	514.34 ± 62.91^cd^	187.67 ± 28.19^c^
Bacitracin zinc	622.81 ± 247.95^c^	499.11 ± 90.44^d^	194.39 ± 24.62^b^
50	900.62 ± 302.23^bc^	675.09 ± 63.65^ab^	233.52 ± 41.42^b^
100	1322.32 ± 282.14^a^	728.07 ± 93.87^a^	283.70 ± 20.15^a^
200	1298.68 ± 158.08^a^	725.94 ± 78.05^a^	246.45 ± 35.73^a^
FSE(mg/kg)	
400	913.59 ± 279.98^bc^	610.40 ± 90.21^ab^	236.18 ± 23.81^b^
800	1081.69 ± 252.54^ab^	588.72 ± 72.17^bc^	233.92 ± 36.79^b^
ANOVA	<0.01	<0.01	<0.01
*P*-value	
Linear	0.02	0.04	0.01
Quadratic	0.01	<0.01	<0.01

*Different lowercase letters with the same column date meant significant difference (P ≤ 0.05)*.

^1^* Each value represents the mean of 6 replicate pens*.

**Table 8:** Effect of fenugreek seed extract on serum cytokine content in broilers^1^.

**Items**	**Basic fodder**	**Bacitracin zinc**	**FSE(mg/kg)**					***P*** **-value**		
			**50**	**100**	**200**	**400**	**800**	**ANOVA**	**Linear**	**Quadratic**
IL-1	165.13 ± 31.55^ab^	130.40 ± 11.92^b^	165.86 ± 34.18^ab^	157.43 ± 44.24^ab^	194.5 ± 30.33^a^	180.05 ± 30.87^a^	150.82 ± 34.84^ab^	0.05	0.23	0.34
IL-2	219.35 ± 20.22	233.53 ± 40.68	228.48 ± 20.48	233.12 ± 23.16	230.87 ± 35.16	211.87 ± 34.58	224.67 ± 38.22	0.88	0.70	0.70
IL-6	19.25 ± 4.00	18.77 ± 4.06	16.36 ± 3.15	14.56 ± 2.36	16.75 ± 4.45	14.76 ± 4.91	18.54 ± 4.57	0.26	0.28	0.07
IL-10	53.88 ± 5.33^ab^	52.01 ± 4.95^b^	50.63 ± 3.85^b^	57.28 ± 5.88^ab^	57.84 ± 4.68^ab^	59.51 ± 6.24^a^	52.46 ± 4.75^b^	0.03	0.17	0.22
IFN-γ	50.20 ± 9.89^bc^	45.52 ± 8.33^c^	53.67 ± 8.62^bc^	62.23 ± 6.90^a^	59.50 ± 7.19^ab^	51.92 ± 3.88^bc^	55.58 ± 6.37^bc^	0.01	0.07	0.03
TNF-α	47.30 ± 4.16	46.35 ± 7.41	40.17 ± 7.62	38.01 ± 6.79	36.67 ± 7.68	43.31 ± 8.73	48.99 ± 14.39	0.11	0.82	0.01

*Different lowercase letters with the same column date meant significant difference (P ≤ 0.05)*.

^1^* Each value represents the mean of 6 replicate pens*.

## Results

### Growth Performance

As shown in Table 4, dietary FSE levels did not significantly affect ADFI (*P* > 0.05). The ADG linearly and quadratically increased (*P* < 0.01) and F/G decreased linearly and quadratically (*P* < 0.01) with increasing FSE supplementation in broilers. The ADG of 50 and 800 mg/kg FSE groups was significantly higher than that of the basic fodder group and bacitracin zinc group (*P* < 0.01). The F/G of 50, 100, 200, 400, 800 mg/kg FSE groups was significantly lower than that of the basic fodder group and bacitracin zinc group (*P* < 0.01).

### Immune Organ Indexes

As shown in Table 5, detary FSE levels did not significantly affect spleen index and bursa of Fabricius index (*P* > 0.05), but the thymus index decreased linearly (*P* < 0.05) with increasing FSE supplementation in broilers.

### Serum Biochemical Indexes

As shown in Table 6, dietary FSE levels did not significantly affect the serum TP,ALB,TG,HDL-C,ALT,AST,BUN,CREA contents (*P* > 0.05). The serum TC content linearly and quadratically decreased with increasing FSE supplementation in broilers (*P* < 0.05). The serum TC content of 50, 100, 400, and 800 mg/kg FSE groups was significantly lower than that of the basic fodder group (*P* < 0.05), and no significant difference from the bacitracin zinc group (*P* > 0.05). The serum LDL-C content of 50, 100, and 800 mg/kg FSE groups was significantly lower than that of the basic fodder group (*P* < 0.05), and no significant difference compared with the bacitracin zinc group (*P* > 0.05). The serum GLU content of 200 and 800 mg/kg FSE groups was significantly higher than that of the basic fodder group (*P* < 0.05).

### Serum Immunoglobulin

As shown in Table 7, the serum IgG, IgM, IgA contents linearly and quadratically increased with increasing FSE supplementation in broilers (*P* < 0.05). The serum IgG content of 100 and 200 mg/kg FSE groups was significantly higher than that of the basic fodder group and bacitracin zinc group (*P* < 0.05). The serum IgG content of 800 mg/kg FSE group was significantly higher than that of the bacitracin zinc group (*P* < 0.05), but no significant difference from the basic fodder group (*P* > 0.05). The serum IgM content of 50, 100, 200, and 400 mg/kg FSE groups was significantly higher than that the basic fodder group and bacitracin zinc group (*P* < 0.05). The serum IgM content of 800 mg/kg FSE group was significantly higher than that of bacitracin zinc group (*P* < 0.05). The serum IgA content of 50, 100, 200, 400, and 800 mg/kg FSE groups was significantly higher than that of the basic fodder group (*P* < 0.05). The serum IgA content of 100 and 200 mg/kg FSE groups was significantly higher than that of the bacitracin zinc group (*P* < 0.05).

### Serum Cytokine

As shown in Table 8, there was no significant difference in the serum IL-2, IL-6, TNF-α content in each group (*P* > 0.05). Contrasted with the bacitracin zinc group, the serum IL-1 content of 200 and 400 mg/kg FSE groups was significantly increased (*P* = 0.05), but not significantly different from the basic fodder group (*P* > 0.05). The serum IL-10 content of 50, 100, 200, and 800 mg/kg FSE groups was not significantly different between that the basic fodder group and bacitracin zinc group (*P* > 0.05). The serum IL-10 content of 400 mg/kg FSE group was significantly higher than that of the bacitracin zinc group (*P* < 0.05), but not significantly different from the basic fodder group (*P* > 0.05). The serum IFN-γ content quadratically increased with increasing FSE supplementation in broilers (*P* < 0.05). The serum TNF-α content quadratically decreased with increasing FSE supplementation in broilers (*P* < 0.05). The serum IFN-γ content of 50, 400, and 800 mg/kg FSE groups was not significantly different compared with the basic fodder group and bacitracin zinc group (*P* > 0.05). The serum IFN-γ content of 100 and 200 mg/kg FSE groups was significantly higher than that of the bacitracin zinc group (*P* < 0.05). The serum IFN-γ content of 100 mg/kg FSE group was significantly higher than that of the basic fodder group (*P* < 0.05).

### The MRNA Expression of TLR4/MyD88/NF-κB Signaling Pathways

As shown in Figure 1, there were no significant differences in the expression levels of TLR4,TRAF6,IFN-γ, IL-1, and IL-10 mRNA in the spleen TLR4/MyD88/ NF-κB signaling pathway of each group (*P* > 0.05). The expression of MyD88 mRNA in the spleen of 50, 400, and 800 mg/kg FSE groups was not significantly different between that of the basic fodder group and bacitracin zinc group (*P* > 0.05). The expression of MyD88 mRNA in the spleen of 100 and 200 mg/kg FSE groups was not significantly different from the basic fodder group (*P* > 0.05), but was significantly lower than that in the bacitracin zinc group (*P* = 0.05). The expression of MyD88 mRNA in the spleen of the bacitracin zinc group was significantly higher than that of the basic fodder group (*P* = 0.05). The expression of NF-κB mRNA in the spleen of 50, 100, 200, and 400 mg/kg FSE groups was not significantly different from the basic fodder group (*P* > 0.05), but was significantly lower than that in the bacitracin zinc group (*P* < 0.01). The expression of NF-κB mRNA in the spleen of 800 mg/kg FSE group was significantly higher than that of the basic fodder group (*P* < 0.05), and was not significantly different with that of bacitracin zinc group (*P* > 0.05). The expression of NF-κB mRNA in the spleen of the bacitracin zinc group was significantly higher than that of the basic fodder group (*P* < 0.01).

## Discussion

Fenugreek contains carbohydrates and fatty acids, which can improve the growth performance of livestock and poultry. Alloui et al. (14) showed that broilers fed a diet containing 3,000 mg/kg FSE could increase broiler feed intake and reduce F/G. Adding 2% fenugreek seed powder to the diet could increase the ADFI of broilers, while adding 3% fenugreek seed powder to the diet reduces the ADFI of broilers (15). Amein et al. (16) and Laudadio et al. (17) found that the powdered seeds of fenugreek and the 0.5% fenugreek in the diet could reduce F/G of broilers. There was a research result showing that greater dietary concentrations of fenugreek reduced BW and increase F/G (18). In this study, we observed that the 50 and 800 mg/kg FSE groups significantly increased ADG, and except 400 mg/kg FSE, other doses of FSE (50–800 mg/kg) significantly increased the final BW of broilers. This may be affected by the number of samples. Because from the numerical point of view, 400 mg/kg FSE also has a tendency to increase the final BW of broilers, and 100–400 mg/kg FSE has a tendency to increase the ADG of broilers. And the final BW and ADG increased linearly and quadratically with increasing FSE supplementation in broilers. Moreover, dietary FSE supplementation significantly decreased F/G. The F/G decreased linearly and quadratically with increasing FSE supplementation in broilers. These results were similar to Amein et al. (16) and Laudadio et al. (17). FSE can improve broiler production, ensuring the health of chickens without toxic side effects to humans.

The main immune organs of the poultry body are the thymus, spleen and bursa of Fabricius. The bursa of Fabricius is extremely important for the maturation of plasma cells and B lymphocytes that can synthesize and secrete antibodies (19). The spleen of poultry is one of the most important peripheral lymphatic organs that respond to foreign antigen stimulation (20). The development of immune organs greatly affects the immune function and disease resistance of the body. Its weight is caused by the growth and division of its own cells. The immune organ index can reflect the development of immune organs and the functional status of immune cells, indirectly reflect the body's immune level (21). Raju and Bird (22) and Bin-Hafeez et al. (8) found that aqueous extracts of fenugreek could increase thymus index. In this experiment, FSE as a feed additive added to broiler feed show no effect on immune organ index, which is inconsistent with the report of Raju and Bin-Hafeez. The variation in the results from different studies might be due to the difference in the rearing environment and species used (22).

Immunoglobulin (Ig) is a kind of macromolecular protein that can bind with antigen and is rapidly produced after the body stimulated by foreign pathogens. It is the most commonly used detection method for the performance of the body's humoral immune function (23). IgM is the first-line defense against a broad range of infections (24). The presence of immunoglobulins especially IgA and IgG in chicken sera enhances the immunity of the birds (25). According to the research reports, FSE can promote Ig secretion in animals. Hossain et al. (26) found that FSE significantly increased IgG levels in growing pigs. In this study, we observed that every level of FSE dietary supplementation had a positive regulation effect on the serum IgG, IgM, and IgA of broilers, and among them 100 mg/kg is the best. It shows that FSE could significantly increase the level of immunoglobulin in broilers, which is beneficial to enhance the immunity of broilers. The main reason why FSE affect immunoglobulin secretion is that FSE contains 50% polysaccharides, and polysaccharide compounds can enhance the immunomodulatory activity of macrophages in animal bodies (27, 28), have the ability to stimulate the production of serum immunoglobulins, and have the potential to regulate innate and adaptive immunity (29, 30).

Serum biochemical indicators can reflect the body's metabolism and some disease states as an effective indicator for checking the health of animal's body. The concentration of TC and TG in serum can be used as indicators of lipid metabolism. Under normal conditions, serum triglycerides maintain dynamic balance. The increase in the concentration of TC and TG in serum indicates that the body's lipid metabolism is abnormal, or the accumulation of fat in the animal's body increases, resulting in an increase in blood lipid contents in the serum (31). LDL-C and HDL-C are lipoproteins associated with cholesterol transport (32). Both are related to atherosclerosis and cardiovascular disease. The former (LDL-C) is positively correlated with the occurrence of the disease, and the latter (HDL-C) is negatively correlated with the occurrence of the disease. FSE can control blood lipids and lower serum total cholesterol. The results of Belguith-Hadriche et al. (33) showed that the use of fenugreek ethyl acetate extracts significantly reduced the levels of TC, TG, and LDL-C in plasma while increasing the plasma levels of HDL-C in plasma. Begum et al. (34) found that FSE has a significant increase in serum HDL-C concentration. In this study, we observed that dietary FSE supplementation decreased the serum TC content in the broiler, and the 50, 100, and 800 mg/kg FSE decreased the serum LDL-C content, and the 200 and 800 mg/kg FSE increased the serum GLU content. Also, FSE had no effect on serum TG and HDL-C. The test results are similar to those of Begum et al. (34) and Belguith-Hadriche et al. (33). The results showed that FSE has the effect on lowering blood lipids, which is beneficial to the body lipid metabolism of broilers. The reason why FSE reduced the content of TC and LDL-C may be related to the alkaloids contained in fenugreek extract (35). According to research, alkaloids can reduce blood TC and LDL-C levels, while beneficially increasing HDL-C levels or no effect on HDL-C levels (36). Trigonelline in FSE may control the absorption of intestinal cholesterol and affect the LDL cholesterol clearance mediated by LDL receptors, thereby controlling the serum cholesterol of broilers, affecting the lipid metabolism of broilers, and playing the role of lowering blood lipids (37–40).

Cytokines are small molecular proteins produced by exogenous or endogenous stimulants induced by immune cells, endothelial cells, and fat cells in the body. They have various physiological functions such as regulating immunity, stimulating endothelial growth, and repairing damage. According to their function and source, cytokines are divided into interferon (IFN), interleukin (IL), tumor necrosis factor (TNF), etc. (41). TNF-α mainly causes white blood cells to accumulate in the inflammation site and has a dual biological role. On the one hand, it is an important medium for the immune protection of the body; on the other hand, it can participate in the immune pathological damage of the body. It plays a very important role in the pathogenesis of tumors and immune diseases (42). IFN-γ has the ability to interfere with virus infection and replication, and its main function is immune regulation (43). The increase of serum IFN-γ is beneficial to activate the body's immunity (44, 45). IL-1, IL-2, and IL-6 are pro-inflammatory factors in the body. Their reduced expression in the body is beneficial to improve the body's anti-inflammatory response and regulate the body's immune function. On the contrary, it can reflect that the body has corresponding inflammation. The reaction will also affect the body's immunity and make the body's immune function imbalance (46, 47). As an anti-inflammatory factor, IL-10 expression in the body is beneficial to improve or activate the body's immune function (48). Studies have shown that FSE could stimulate the regulation of cytokines and significantly reduce the levels of IL-1α, IL-1β, IL-2, IL-6, and TNF-α (49–52). In this study, compared with the bacitracin zinc group, the 200 and 400 mg/kg FSE dietary supplementation increased the serum IL-1 content, the 400 mg/kg FSE increased the serum IL-10 content, the 100 and 200 mg/kg FSE increased the serum IFN-γ content. The serum IFN-γ content quadratically increased with increasing FSE supplementation in broilers. The serum TNF-α content quadratically decreased with increasing FSE supplementation in broilers. The results of this test showed that FSE can increase the secretion of anti-inflammatory factors in broilers and inhibit the synthesis of pro-inflammatory factors, which is basically similar with the studies of Tripathi et al. (10) and Ghosh et al. (51). Fenugreek contains a number of important, beneficial flavonoids, and polyphenol compounds. Nagulapalli et al. (5) have reported the presence of a wide range of flavonoids, namely quercetin, luteolin, vitexin, and 7, 4′-dimethoxy flavanones in the alcoholic extracts of the whole plant. Benayad et al. (53) have reported similar findings of the presence of aglycones kaempferol, quercetin, tricin, and naringenin. The reason why FSE increases serum IFN-γ and IL-10 may be due to the anti-inflammatory active substances contained in FSE. For example, fenugreek flavonoids are the main anti-inflammatory and antioxidant bioactive components (54, 55), and fenugreek polysaccharides are also have anti-inflammatory properties (56). FSE increased the serum IFN-γ value of broiler chickens, may regulate Th17 and Th1 cells (57), inhibit the secretion of pro-inflammatory factors (IL-1, IL-2, IL-6, TNF-α), and promote the anti-inflammatory factor IL-10 secretion, thereby activating the immune function of broilers. In addition, this test found that the serum levels of IL-1, IFN-γ, and IL-10 in the zinc bacitracin group were reduced, which inhibited the activation of inflammatory and immune responses to a certain extent.

Toll-like receptors 4 (TLR4) is the earliest discovered protein among TLR-related proteins. It is distributed in almost all cell lines and is expressed in cells involved in host defense functions (58). The mechanism of its pathway is that the TLR4 receptor binds to the corresponding ligand, the signal is transduced to the TLR4 region, and then the NF-κB and MAPK signaling pathways are further activated to promote the expression and activation of various inflammatory cytokine genes (59). MyD88 is both a key downstream signaling ligand of TLR4 and an important protein in the NF-κB signaling pathway (60, 61). Their mechanism of action is that some extracellular inflammatory signals are presented to MyD88 through the TLR domain, allowing MyD88 to interact with interleukin-1 receptor-associated kinase 4 (IRAK4) to phosphorylate IRAK4 (62). Then phosphorylate IRAK1, and then act on ubiquitin ligase (TRAF6). After MyD88 is activated, TAK-1 and IκB kinase (IKK) are activated under the action of trans-growth factor-β activated kinase 1 binding protein (TAB-1/TAB-2) to activate transcription factors, and then initiate signal transduction through the nucleus (63). Research has shown that plant polysaccharides and plant flavonoids can activate the immune system and play an immunomodulatory effect (64, 65). Hou et al. (64) found that G. frondosa polysaccharide activated macrophages through TLR4-MyD88-IKKβ-NF-κBp65 signaling pathways. Juglan, a flavonol derivative, in LPS-induced C57B/L6 mice significantly reduced pro-inflammatory cytokines and blocked TLR4/NF-κB pathway (66). However, in the present study, the mRNA expression of TLR4/MyD88/NF-κB signaling pathways was not significantly affected by lower doses (0–400 mg/kg) of FSE containing 50% polysaccharides and 10% flavonoids. The variation in the results from different studies might be due to the different species used and the way of adding active substances (64, 66). Furthermore, in the present study, the 800 mg/kg FSE treatment group up-regulated NF-κB expression. The zinc bacitracin group significantly increased the expression levels of MyD88 and NF-κB mRNA in the spleen of broilers. It showed that bacitracin zinc and high doses (800 mg/kg) of FSE have some negative effects on body immunity by the TLR4/MyD88/ NF-κB signaling pathway.

## Conclusion

Dietary FSE could further improve growth performance, promote the secretion of broiler immunoglobulins, regulate the body's cytokines and have a positive effect on immunity. FSE is expected to replace antibiotics to improve broiler health and growth and increase chicken production while ensuring chicken quality and health. The optimal concentration of 100 mg/kg for FSE has been initially found.

## Data Availability Statement

The original contributions presented in the study are included in the article/Supplementary Material, further inquiries can be directed to the corresponding author/s.

## Ethics Statement

The animal study was reviewed and approved by the Institutional Animal Care and Use Committee of Hunan Agricultural University, Hunan, China.

## Author Contributions

All authors listed have made a substantial, direct, and intellectual contribution to the work and approved it for publication.

## Funding

This project was funded by National Natural Science Foundation of China (31872991), National Natural Science Foundation of Hunan Province-China (2020JJ4364), and Project of Science and Technology Department of Hunan Province (2020NK4247). Cooperation project between Hunan Agricultural University and Hunan Jinhan Pharmaceutical Co., Ltd. (2019xny-js065).

## Conflict of Interest

YZ was employed by Geneham Pharmaceutical Co., Ltd. SZ was employed by Hunan Wenfeng Agricultural and Animal Husbandry Technology Co. The remaining authors declare that the research was conducted in the absence of any commercial or financial relationships that could be construed as a potential conflict of interest.

## Publisher's Note

All claims expressed in this article are solely those of the authors and do not necessarily represent those of their affiliated organizations, or those of the publisher, the editors and the reviewers. Any product that may be evaluated in this article, or claim that may be made by its manufacturer, is not guaranteed or endorsed by the publisher.

## Abbreviations

FSE: Fenugreek seed extract
ADG: Average daily gain
ADFI: Average daily feed intake
F/G: Feed/Gain
TP: Total protein
ALB: Albumin
TG: Triglyceride
TC: Total cholesterol
HDL-C: High density lipoprotein cholesterol
LDL-C: Low density lipoprotein-cholesterol
GLU: Glucose
ALT: Alanine aminotransferase
AST: Aspartate aminotransferase
BUN: Blood urea nitrogen
CREA: Creatinine
IL-1: Interleukin-1
IL-2: Interleukin-2
IL-6: Interleukin-6
IL-10: Interleukin-10
IFN-γ: Interferon
TNF-α: Tumor necrosis factor
IgG: Immunoglobulin G
IgM: Immunoglobulin M
IgA: Immunoglobulin A
TLR4: Toll-like receptors 4
GAPDH: Glyceraldehyde-3-phosphate dehydrogenase
MyD88: Myeloid differentiation factor 88
TRAF6: TNF receptor associated factor 6
NF-κB: Nuclear factor-κb
IL-1β: Interleukin-1β
MAPK: Mitogen-activated protein kinase
IRAK4: Interleukin-1 receptor-associated kinase 4
TAK-1: Transforming growth factor-β activated protein kinase
IKK: IκB kinase
IRAK4: Interleukin-1 receptor-associated kinase 4
ELISA: Enzyme-linked immunosorbent assay
TAB-1: Transforming growth factor-β activated protein kinase binding protein.
